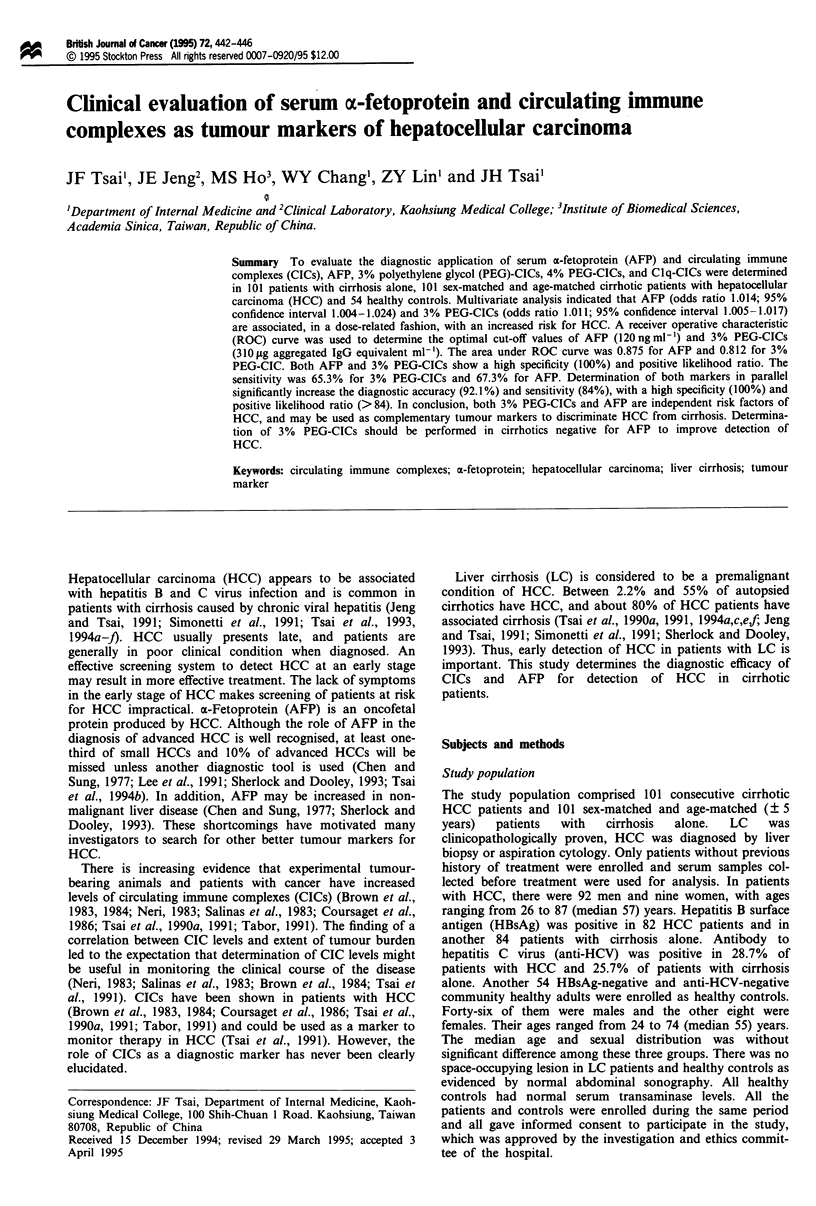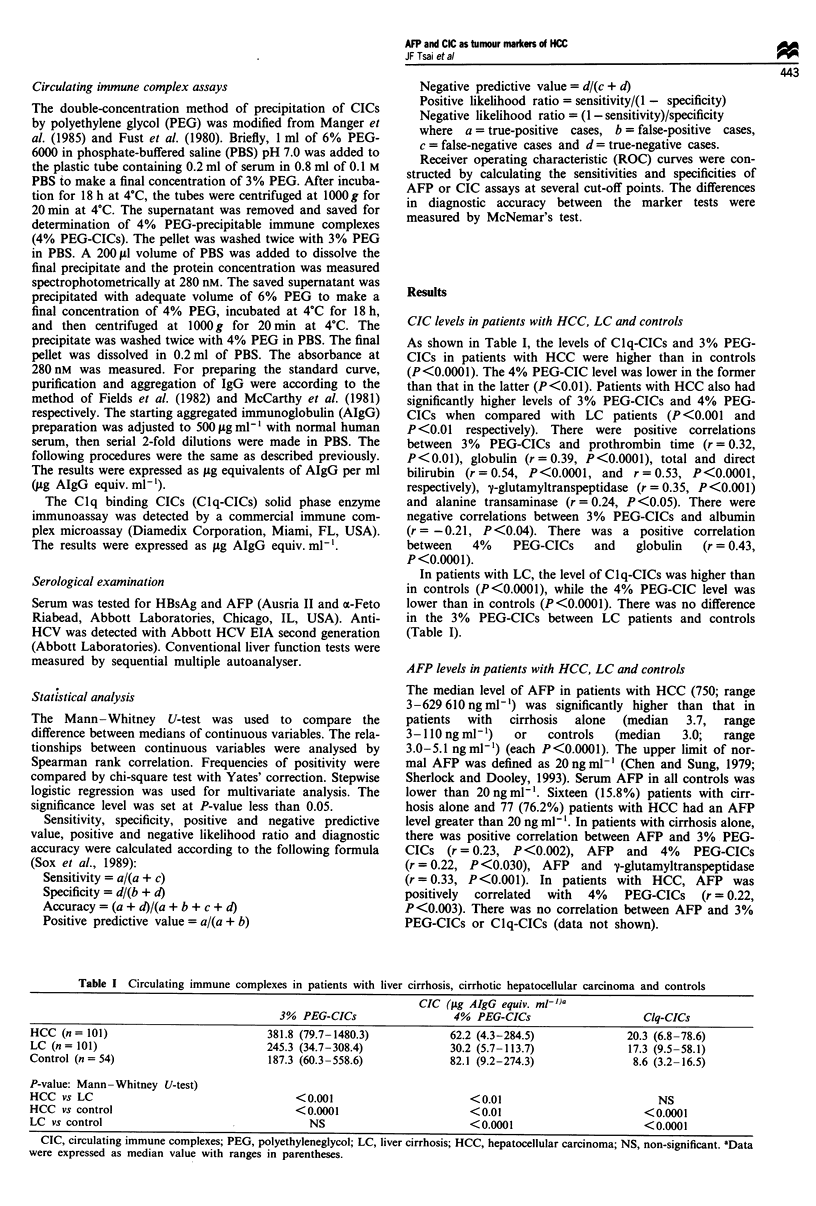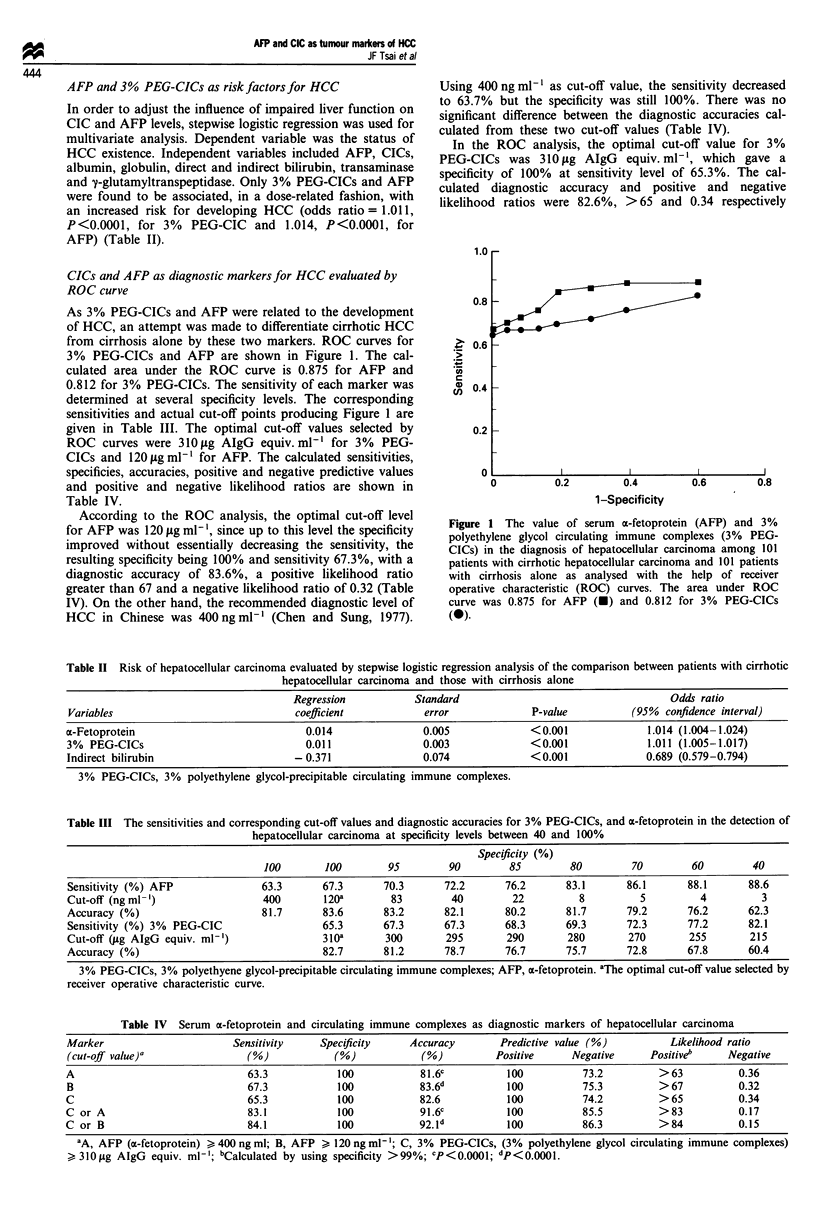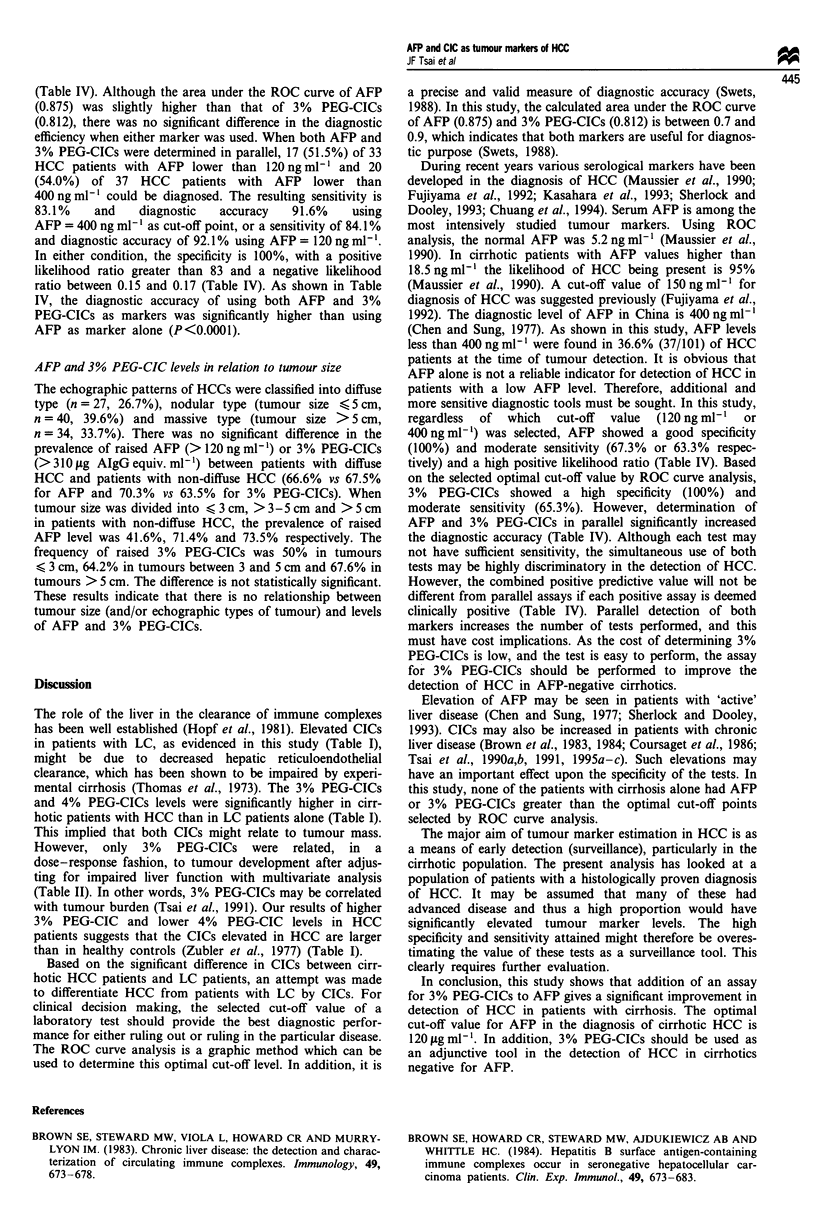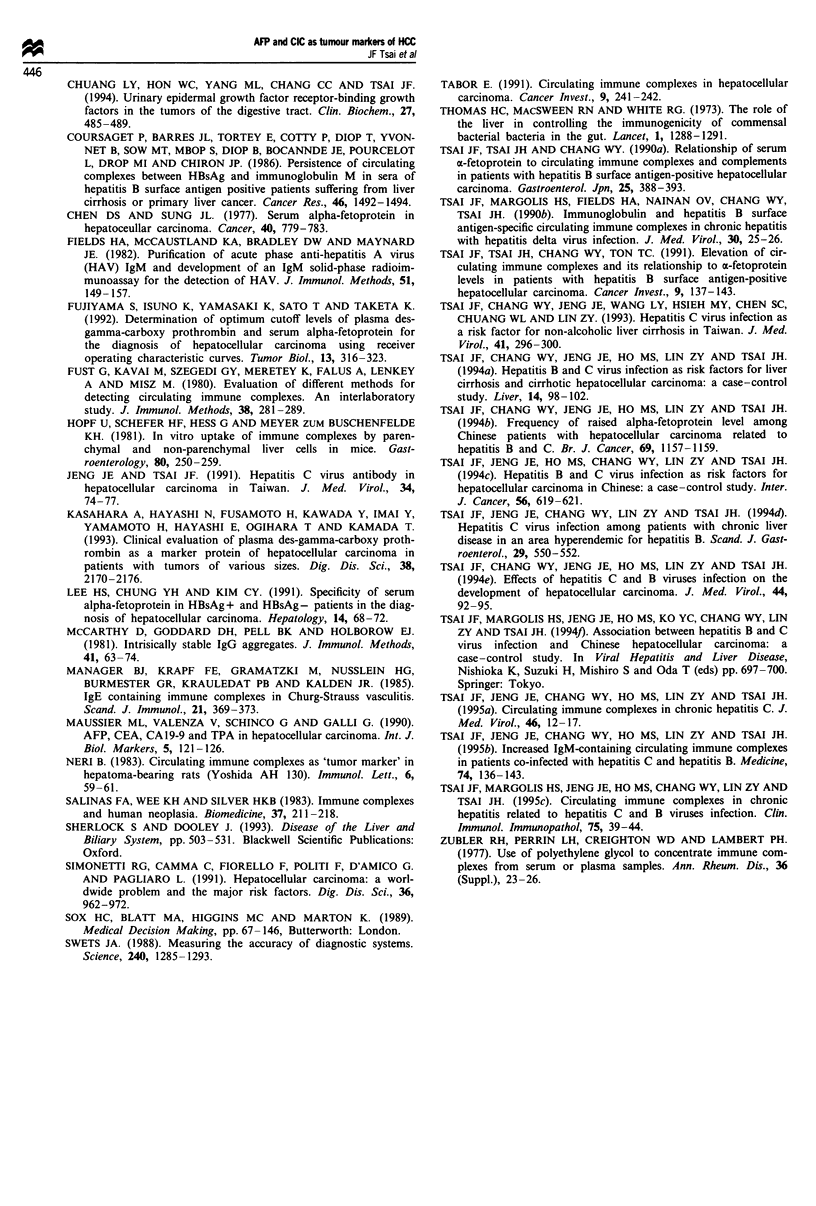# Clinical evaluation of serum alpha-fetoprotein and circulating immune complexes as tumour markers of hepatocellular carcinoma.

**DOI:** 10.1038/bjc.1995.352

**Published:** 1995-08

**Authors:** J. F. Tsai, J. E. Jeng, M. S. Ho, W. Y. Chang, Z. Y. Lin, J. H. Tsai

**Affiliations:** Department of Internal Medicine, Kaohsiung Medical College, Taiwan, Republic of China.

## Abstract

To evaluate the diagnostic application of serum alpha-fetoprotein (AFP) and circulating immune complexes (CICs), AFP, 3% polyethylene glycol (PEG)-CICs, 4% PEG-CICs, and C1q-CICs were determined in 101 patients with cirrhosis alone, 101 sex-matched and age-matched cirrhotic patients with hepatocellular carcinoma (HCC) and 54 healthy controls. Multivariate analysis indicated that AFP (odds ratio 1.014; 95% confidence interval 1.004-1.024) and 3% PEG-CICs (odds ratio 1.011; 95% confidence interval 1.005-1.017) are associated, in a dose-related fashion, with an increased risk for HCC. A receiver operative characteristic (ROC) curve was used to determine the optimal cut-off values of AFP (120 ng ml-1) and 3% PEG-CICs (310 micrograms aggregated IgG equivalent ml-1). The area under ROC curve was 0.875 for AFP and 0.812 for 3% PEG-CIC. Both AFP and 3% PEG-CICs show a high specificity (100%) and positive likelihood ratio. The sensitivity was 65.3% for 3% PEG-CICs and 67.3% for AFP. Determination of both markers in parallel significantly increase the diagnostic accuracy (92.1%) and sensitivity (84%), with a high specificity (100%) and positive likelihood ratio (> 84). In conclusion, both 3% PEG-CICs and AFP are independent risk factors of HCC, and may be used as complementary tumour markers to discriminate HCC from cirrhosis. Determination of 3% PEG-CICs should be performed in cirrhotics negative for AFP to improve detection of HCC.


British Journal of Cancer (1995) 72, 442-446
© 1995 Stockton Press All rights reserved 0007-0920/95 $12.00

### Clinical evaluation of serum α-fetoprotein and circulating immune complexes as tumour markers of hepatocellular carcinoma

JF Tsai[1], JE Jeng[2], MS Ho[3], WY Chang[1], ZY Lin[1] and JH Tsai[1]

[1]*Department of Internal Medicine and* [2]*Clinical Laboratory, Kaohsiung Medical College;* [3]*Institute of Biomedical Sciences, Academia Sinica, Taiwan, Republic of China.*

**Summary** To evaluate the diagnostic application of serum α-fetoprotein (AFP) and circulating immune complexes (CICs), AFP, 3% polyethylene glycol (PEG)-CICs, 4% PEG-CICs, and C1q-CICs were determined in 101 patients with cirrhosis alone, 101 sex-matched and age-matched cirrhotic patients with hepatocellular carcinoma (HCC) and 54 healthy controls. Multivariate analysis indicated that AFP (odds ratio 1.014; 95% confidence interval 1.004-1.024) and 3% PEG-CICs (odds ratio 1.011; 95% confidence interval 1.005-1.017) are associated, in a dose-related fashion, with an increased risk for HCC. A receiver operative characteristic (ROC) curve was used to determine the optimal cut-off values of AFP (120 ng $ml^{-1}$) and 3% PEG-CICs (310 μg aggregated IgG equivalent $ml^{-1}$). The area under ROC curve was 0.875 for AFP and 0.812 for 3% PEG-CIC. Both AFP and 3% PEG-CICs show a high specificity (100%) and positive likelihood ratio. The sensitivity was 65.3% for 3% PEG-CICs and 67.3% for AFP. Determination of both markers in parallel significantly increase the diagnostic accuracy (92.1%) and sensitivity (84%), with a high specificity (100%) and positive likelihood ratio (> 84). In conclusion, both 3% PEG-CICs and AFP are independent risk factors of HCC, and may be used as complementary tumour markers to discriminate HCC from cirrhosis. Determination of 3% PEG-CICs should be performed in cirrhotics negative for AFP to improve detection of HCC.

**Keywords:** circulating immune complexes; α-fetoprotein; hepatocellular carcinoma; liver cirrhosis; tumour marker

Hepatocellular carcinoma (HCC) appears to be associated with hepatitis B and C virus infection and is common in patients with cirrhosis caused by chronic viral hepatitis (Jeng and Tsai, 1991; Simonetti *et al.*, 1991; Tsai *et al.*, 1993, 1994*a–f*). HCC usually presents late, and patients are generally in poor clinical condition when diagnosed. An effective screening system to detect HCC at an early stage may result in more effective treatment. The lack of symptoms in the early stage of HCC makes screening of patients at risk for HCC impractical. α-Fetoprotein (AFP) is an oncofetal protein produced by HCC. Although the role of AFP in the diagnosis of advanced HCC is well recognised, at least one-third of small HCCs and 10% of advanced HCCs will be missed unless another diagnostic tool is used (Chen and Sung, 1977; Lee *et al.*, 1991; Sherlock and Dooley, 1993; Tsai *et al.*, 1994*b*). In addition, AFP may be increased in non-malignant liver disease (Chen and Sung, 1977; Sherlock and Dooley, 1993). These shortcomings have motivated many investigators to search for other better tumour markers for HCC.

There is increasing evidence that experimental tumour-bearing animals and patients with cancer have increased levels of circulating immune complexes (CICs) (Brown *et al.*, 1983, 1984; Neri, 1983; Salinas *et al.*, 1983; Coursaget *et al.*, 1986; Tsai *et al.*, 1990*a*, 1991; Tabor, 1991). The finding of a correlation between CIC levels and extent of tumour burden led to the expectation that determination of CIC levels might be useful in monitoring the clinical course of the disease (Neri, 1983; Salinas *et al.*, 1983; Brown *et al.*, 1984; Tsai *et al.*, 1991). CICs have been shown in patients with HCC (Brown *et al.*, 1983, 1984; Coursaget *et al.*, 1986; Tsai *et al.*, 1990*a*, 1991; Tabor, 1991) and could be used as a marker to monitor therapy in HCC (Tsai *et al.*, 1991). However, the role of CICs as a diagnostic marker has never been clearly elucidated.

Correspondence: JF Tsai, Department of Internal Medicine, Kaohsiung Medical College, 100 Shih-Chuan 1 Road. Kaohsiung, Taiwan 80708, Republic of China
Received 15 December 1994; revised 29 March 1995; accepted 3 April 1995

Liver cirrhosis (LC) is considered to be a premalignant condition of HCC. Between 2.2% and 55% of autopsied cirrhotics have HCC, and about 80% of HCC patients have associated cirrhosis (Tsai *et al.*, 1990*a*, 1991, 1994*a,c,e,f*; Jeng and Tsai, 1991; Simonetti *et al.*, 1991; Sherlock and Dooley, 1993). Thus, early detection of HCC in patients with LC is important. This study determines the diagnostic efficacy of CICs and AFP for detection of HCC in cirrhotic patients.

### Subjects and methods

### *Study population*

The study population comprised 101 consecutive cirrhotic HCC patients and 101 sex-matched and age-matched (± 5 years) patients with cirrhosis alone. LC was clinicopathologically proven, HCC was diagnosed by liver biopsy or aspiration cytology. Only patients without previous history of treatment were enrolled and serum samples collected before treatment were used for analysis. In patients with HCC, there were 92 men and nine women, with ages ranging from 26 to 87 (median 57) years. Hepatitis B surface antigen (HBsAg) was positive in 82 HCC patients and in another 84 patients with cirrhosis alone. Antibody to hepatitis C virus (anti-HCV) was positive in 28.7% of patients with HCC and 25.7% of patients with cirrhosis alone. Another 54 HBsAg-negative and anti-HCV-negative community healthy adults were enrolled as healthy controls. Forty-six of them were males and the other eight were females. Their ages ranged from 24 to 74 (median 55) years. The median age and sexual distribution was without significant difference among these three groups. There was no space-occupying lesion in LC patients and healthy controls as evidenced by normal abdominal sonography. All healthy controls had normal serum transaminase levels. All the patients and controls were enrolled during the same period and all gave informed consent to participate in the study, which was approved by the investigation and ethics committee of the hospital.

*Circulating immune complex assays*

The double-concentration method of precipitation of CICs by polyethylene glycol (PEG) was modified from Manger *et al.* (1985) and Fust *et al.* (1980). Briefly, 1 ml of 6% PEG-6000 in phosphate-buffered saline (PBS) pH 7.0 was added to the plastic tube containing 0.2 ml of serum in 0.8 ml of 0.1 M PBS to make a final concentration of 3% PEG. After incubation for 18 h at 4°C, the tubes were centrifuged at 1000 *g* for 20 min at 4°C. The supernatant was removed and saved for determination of 4% PEG-precipitable immune complexes (4% PEG-CICs). The pellet was washed twice with 3% PEG in PBS. A 200 µl volume of PBS was added to dissolve the final precipitate and the protein concentration was measured spectrophotometrically at 280 nM. The saved supernatant was precipitated with adequate volume of 6% PEG to make a final concentration of 4% PEG, incubated at 4°C for 18 h, and then centrifuged at 1000 *g* for 20 min at 4°C. The precipitate was washed twice with 4% PEG in PBS. The final pellet was dissolved in 0.2 ml of PBS. The absorbance at 280 nM was measured. For preparing the standard curve, purification and aggregation of IgG were according to the method of Fields *et al.* (1982) and McCarthy *et al.* (1981) respectively. The starting aggregated immunoglobulin (AIgG) preparation was adjusted to 500 µg $ml^{-1}$ with normal human serum, then serial 2-fold dilutions were made in PBS. The following procedures were the same as described previously. The results were expressed as µg equivalents of AIgG per ml (µg AIgG equiv. $ml^{-1}$).

The Clq binding CICs (Clq-CICs) solid phase enzyme immunoassay was detected by a commercial immune complex microassay (Diamedix Corporation, Miami, FL, USA). The results were expressed as µg AIgG equiv. $ml^{-1}$.

*Serological examination*

Serum was tested for HBsAg and AFP (Ausria II and α-Feto Riabead, Abbott Laboratories, Chicago, IL, USA). Anti-HCV was detected with Abbott HCV EIA second generation (Abbott Laboratories). Conventional liver function tests were measured by sequential multiple autoanalyser.

*Statistical analysis*

The Mann–Whitney *U*-test was used to compare the difference between medians of continuous variables. The relationships between continuous variables were analysed by Spearman rank correlation. Frequencies of positivity were compared by chi-square test with Yates' correction. Stepwise logistic regression was used for multivariate analysis. The significance level was set at *P*-value less than 0.05.

Sensitivity, specificity, positive and negative predictive value, positive and negative likelihood ratio and diagnostic accuracy were calculated according to the following formula (Sox *et al.*, 1989):

Sensitivity = $a/(a + c)$
Specificity = $d/(b + d)$
Accuracy = $(a + d)/(a + b + c + d)$
Positive predictive value = $a/(a + b)$
Negative predictive value = $d/(c + d)$
Positive likelihood ratio = sensitivity/(1 − specificity)
Negative likelihood ratio = (1 − sensitivity)/specificity
where $a$ = true-positive cases, $b$ = false-positive cases, $c$ = false-negative cases and $d$ = true-negative cases.

Receiver operating characteristic (ROC) curves were constructed by calculating the sensitivities and specificities of AFP or CIC assays at several cut-off points. The differences in diagnostic accuracy between the marker tests were measured by McNemar's test.

### Results

*CIC levels in patients with HCC, LC and controls*

As shown in Table I, the levels of Clq-CICs and 3% PEG-CICs in patients with HCC were higher than in controls ($P<0.0001$). The 4% PEG-CIC level was lower in the former than that in the latter ($P<0.01$). Patients with HCC also had significantly higher levels of 3% PEG-CICs and 4% PEG-CICs when compared with LC patients ($P<0.001$ and $P<0.01$ respectively). There were positive correlations between 3% PEG-CICs and prothrombin time ($r = 0.32$, $P<0.01$), globulin ($r = 0.39$, $P<0.0001$), total and direct bilirubin ($r = 0.54$, $P<0.0001$, and $r = 0.53$, $P<0.0001$, respectively), γ-glutamyltranspeptidase ($r = 0.35$, $P<0.001$) and alanine transaminase ($r = 0.24$, $P<0.05$). There were negative correlations between 3% PEG-CICs and albumin ($r = -0.21$, $P<0.04$). There was a positive correlation between 4% PEG-CICs and globulin ($r = 0.43$, $P<0.0001$).

In patients with LC, the level of Clq-CICs was higher than in controls ($P<0.0001$), while the 4% PEG-CIC level was lower than in controls ($P<0.0001$). There was no difference in the 3% PEG-CICs between LC patients and controls (Table I).

*AFP levels in patients with HCC, LC and controls*

The median level of AFP in patients with HCC (750; range 3–629 610 ng $ml^{-1}$) was significantly higher than that in patients with cirrhosis alone (median 3.7, range 3–110 ng $ml^{-1}$) or controls (median 3.0; range 3.0–5.1 ng $ml^{-1}$) (each $P<0.0001$). The upper limit of normal AFP was defined as 20 ng $ml^{-1}$ (Chen and Sung, 1979; Sherlock and Dooley, 1993). Serum AFP in all controls was lower than 20 ng $ml^{-1}$. Sixteen (15.8%) patients with cirrhosis alone and 77 (76.2%) patients with HCC had an AFP level greater than 20 ng $ml^{-1}$. In patients with cirrhosis alone, there was positive correlation between AFP and 3% PEG-CICs ($r = 0.23$, $P<0.002$), AFP and 4% PEG-CICs ($r = 0.22$, $P<0.030$), AFP and γ-glutamyltranspeptidase ($r = 0.33$, $P<0.001$). In patients with HCC, AFP was positively correlated with 4% PEG-CICs ($r = 0.22$, $P<0.003$). There was no correlation between AFP and 3% PEG-CICs or Clq-CICs (data not shown).

**Table I** Circulating immune complexes in patients with liver cirrhosis, cirrhotic hepatocellular carcinoma and controls

| | CIC (µg AIgG equiv. $ml^{-1}$)[a] | | |
|---|---|---|---|
| | *3% PEG-CICs* | *4% PEG-CICs* | *Clq-CICs* |
| HCC ($n$ = 101) | 381.8 (79.7–1480.3) | 62.2 (4.3–284.5) | 20.3 (6.8–78.6) |
| LC ($n$ = 101) | 245.3 (34.7–308.4) | 30.2 (5.7–113.7) | 17.3 (9.5–58.1) |
| Control ($n$ = 54) | 187.3 (60.3–558.6) | 82.1 (9.2–274.3) | 8.6 (3.2–16.5) |
| *P*-value: Mann–Whitney *U*-test) | | | |
| HCC *vs* LC | <0.001 | <0.01 | NS |
| HCC *vs* control | <0.0001 | <0.01 | <0.0001 |
| LC *vs* control | NS | <0.0001 | <0.0001 |

CIC, circulating immune complexes; PEG, polyethyleneglycol; LC, liver cirrhosis; HCC, hepatocellular carcinoma; NS, non-significant. [a]Data were expressed as median value with ranges in parentheses.

### *AFP and 3% PEG-CICs as risk factors for HCC*

In order to adjust the influence of impaired liver function on CIC and AFP levels, stepwise logistic regression was used for multivariate analysis. Dependent variable was the status of HCC existence. Independent variables included AFP, CICs, albumin, globulin, direct and indirect bilirubin, transaminase and γ-glutamyltranspeptidase. Only 3% PEG-CICs and AFP were found to be associated, in a dose-related fashion, with an increased risk for developing HCC (odds ratio = 1.011, $P<0.0001$, for 3% PEG-CIC and 1.014, $P<0.0001$, for AFP) (Table II).

### *CICs and AFP as diagnostic markers for HCC evaluated by ROC curve*

As 3% PEG-CICs and AFP were related to the development of HCC, an attempt was made to differentiate cirrhotic HCC from cirrhosis alone by these two markers. ROC curves for 3% PEG-CICs and AFP are shown in Figure 1. The calculated area under the ROC curve is 0.875 for AFP and 0.812 for 3% PEG-CICs. The sensitivity of each marker was determined at several specificity levels. The corresponding sensitivities and actual cut-off points producing Figure 1 are given in Table III. The optimal cut-off values selected by ROC curves were 310 μg AIgG equiv. $ml^{-1}$ for 3% PEG-CICs and 120 μg $ml^{-1}$ for AFP. The calculated sensitivities, specificies, accuracies, positive and negative predictive values and positive and negative likelihood ratios are shown in Table IV.

According to the ROC analysis, the optimal cut-off level for AFP was 120 μg $ml^{-1}$, since up to this level the specificity improved without essentially decreasing the sensitivity, the resulting specificity being 100% and sensitivity 67.3%, with a diagnostic accuracy of 83.6%, a positive likelihood ratio greater than 67 and a negative likelihood ratio of 0.32 (Table IV). On the other hand, the recommended diagnostic level of HCC in Chinese was 400 ng $ml^{-1}$ (Chen and Sung, 1977). Using 400 ng $ml^{-1}$ as cut-off value, the sensitivity decreased to 63.7% but the specificity was still 100%. There was no significant difference between the diagnostic accuracies calculated from these two cut-off values (Table IV).

In the ROC analysis, the optimal cut-off value for 3% PEG-CICs was 310 μg AIgG equiv. $ml^{-1}$, which gave a specificity of 100% at sensitivity level of 65.3%. The calculated diagnostic accuracy and positive and negative likelihood ratios were 82.6%, >65 and 0.34 respectively

**Figure 1** The value of serum α-fetoprotein (AFP) and 3% polyethylene glycol circulating immune complexes (3% PEG-CICs) in the diagnosis of hepatocellular carcinoma among 101 patients with cirrhotic hepatocellular carcinoma and 101 patients with cirrhosis alone as analysed with the help of receiver operative characteristic (ROC) curves. The area under ROC curve was 0.875 for AFP (■) and 0.812 for 3% PEG-CICs (●).

**Table II** Risk of hepatocellular carcinoma evaluated by stepwise logistic regression analysis of the comparison between patients with cirrhotic hepatocellular carcinoma and those with cirrhosis alone

| *Variables* | *Regression coefficient* | *Standard error* | P-*value* | *Odds ratio (95% confidence interval)* |
|---|---|---|---|---|
| α-Fetoprotein | 0.014 | 0.005 | <0.001 | 1.014 (1.004–1.024) |
| 3% PEG-CICs | 0.011 | 0.003 | <0.001 | 1.011 (1.005–1.017) |
| Indirect bilirubin | – 0.371 | 0.074 | <0.001 | 0.689 (0.579–0.794) |

3% PEG-CICs, 3% polyethylene glycol-precipitable circulating immune complexes.

**Table III** The sensitivities and corresponding cut-off values and diagnostic accuracies for 3% PEG-CICs, and α-fetoprotein in the detection of hepatocellular carcinoma at specificity levels between 40 and 100%

| | *Specificity (%)* | | | | | | | | |
|---|---|---|---|---|---|---|---|---|---|
| | *100* | *100* | *95* | *90* | *85* | *80* | *70* | *60* | *40* |
| Sensitivity (%) AFP | 63.3 | 67.3 | 70.3 | 72.2 | 76.2 | 83.1 | 86.1 | 88.1 | 88.6 |
| Cut-off (ng $ml^{-1}$) | 400 | 120[a] | 83 | 40 | 22 | 8 | 5 | 4 | 3 |
| Accuracy (%) | 81.7 | 83.6 | 83.2 | 82.1 | 80.2 | 81.7 | 79.2 | 76.2 | 62.3 |
| Sensitivity (%) 3% PEG-CIC | | 65.3 | 67.3 | 67.3 | 68.3 | 69.3 | 72.3 | 77.2 | 82.1 |
| Cut-off (μg AIgG equiv. $ml^{-1}$) | | 310[a] | 300 | 295 | 290 | 280 | 270 | 255 | 215 |
| Accuracy (%) | | 82.7 | 81.2 | 78.7 | 76.7 | 75.7 | 72.8 | 67.8 | 60.4 |

3% PEG-CICs, 3% polyethyene glycol-precipitable circulating immune complexes; AFP, α-fetoprotein. [a]The optimal cut-off value selected by receiver operative characteristic curve.

**Table IV** Serum α-fetoprotein and circulating immune complexes as diagnostic markers of hepatocellular carcinoma

| *Marker (cut-off value)*[a] | *Sensitivity (%)* | *Specificity (%)* | *Accuracy (%)* | *Predictive value (%) Positive* | *Predictive value (%) Negative* | *Likelihood ratio Positive*[b] | *Likelihood ratio Negative* |
|---|---|---|---|---|---|---|---|
| A | 63.3 | 100 | 81.6[c] | 100 | 73.2 | >63 | 0.36 |
| B | 67.3 | 100 | 83.6[d] | 100 | 75.3 | >67 | 0.32 |
| C | 65.3 | 100 | 82.6 | 100 | 74.2 | >65 | 0.34 |
| C or A | 83.1 | 100 | 91.6[c] | 100 | 85.5 | >83 | 0.17 |
| C or B | 84.1 | 100 | 92.1[d] | 100 | 86.3 | >84 | 0.15 |

[a]A, AFP (α-fetoprotein) ⩾ 400 ng ml; B, AFP ⩾ 120 ng $ml^{-1}$; C, 3% PEG-CICs, (3% polyethylene glycol circulating immune complexes) ⩾ 310 μg AIgG equiv. $ml^{-1}$; [b]Calculated by using specificity >99%; [c]$P<0.0001$; [d]$P<0.0001$.

(Table IV). Although the area under the ROC curve of AFP (0.875) was slightly higher than that of 3% PEG-CICs (0.812), there was no significant difference in the diagnostic efficiency when either marker was used. When both AFP and 3% PEG-CICs were determined in parallel, 17 (51.5%) of 33 HCC patients with AFP lower than 120 ng ml$^{-1}$ and 20 (54.0%) of 37 HCC patients with AFP lower than 400 ng ml$^{-1}$ could be diagnosed. The resulting sensitivity is 83.1% and diagnostic accuracy 91.6% using AFP = 400 ng ml$^{-1}$ as cut-off point, or a sensitivity of 84.1% and diagnostic accuracy of 92.1% using AFP = 120 ng ml$^{-1}$. In either condition, the specificity is 100%, with a positive likelihood ratio greater than 83 and a negative likelihood ratio between 0.15 and 0.17 (Table IV). As shown in Table IV, the diagnostic accuracy of using both AFP and 3% PEG-CICs as markers was significantly higher than using AFP as marker alone ($P<0.0001$).

*AFP and 3% PEG-CIC levels in relation to tumour size*

The echographic patterns of HCCs were classified into diffuse type ($n = 27$, 26.7%), nodular type (tumour size $\leqslant 5$ cm, $n = 40$, 39.6%) and massive type (tumour size $>5$ cm, $n = 34$, 33.7%). There was no significant difference in the prevalence of raised AFP ($>120$ ng ml$^{-1}$) or 3% PEG-CICs ($>310$ µg AIgG equiv. ml$^{-1}$) between patients with diffuse HCC and patients with non-diffuse HCC (66.6% *vs* 67.5% for AFP and 70.3% *vs* 63.5% for 3% PEG-CICs). When tumour size was divided into $\leqslant 3$ cm, $>3-5$ cm and $>5$ cm in patients with non-diffuse HCC, the prevalence of raised AFP level was 41.6%, 71.4% and 73.5% respectively. The frequency of raised 3% PEG-CICs was 50% in tumours $\leqslant 3$ cm, 64.2% in tumours between 3 and 5 cm and 67.6% in tumours $>5$ cm. The difference is not statistically significant. These results indicate that there is no relationship between tumour size (and/or echographic types of tumour) and levels of AFP and 3% PEG-CICs.

### Discussion

The role of the liver in the clearance of immune complexes has been well established (Hopf *et al.*, 1981). Elevated CICs in patients with LC, as evidenced in this study (Table I), might be due to decreased hepatic reticuloendothelial clearance, which has been shown to be impaired by experimental cirrhosis (Thomas *et al.*, 1973). The 3% PEG-CICs and 4% PEG-CICs levels were significantly higher in cirrhotic patients with HCC than in LC patients alone (Table I). This implied that both CICs might relate to tumour mass. However, only 3% PEG-CICs were related, in a dose–response fashion, to tumour development after adjusting for impaired liver function with multivariate analysis (Table II). In other words, 3% PEG-CICs may be correlated with tumour burden (Tsai *et al.*, 1991). Our results of higher 3% PEG-CIC and lower 4% PEG-CIC levels in HCC patients suggests that the CICs elevated in HCC are larger than in healthy controls (Zubler *et al.*, 1977) (Table I).

Based on the significant difference in CICs between cirrhotic HCC patients and LC patients, an attempt was made to differentiate HCC from patients with LC by CICs. For clinical decision making, the selected cut-off value of a laboratory test should provide the best diagnostic performance for either ruling out or ruling in the particular disease. The ROC curve analysis is a graphic method which can be used to determine this optimal cut-off level. In addition, it is a precise and valid measure of diagnostic accuracy (Swets, 1988). In this study, the calculated area under the ROC curve of AFP (0.875) and 3% PEG-CICs (0.812) is between 0.7 and 0.9, which indicates that both markers are useful for diagnostic purpose (Swets, 1988).

During recent years various serological markers have been developed in the diagnosis of HCC (Maussier *et al.*, 1990; Fujiyama *et al.*, 1992; Kasahara *et al.*, 1993; Sherlock and Dooley, 1993; Chuang *et al.*, 1994). Serum AFP is among the most intensively studied tumour markers. Using ROC analysis, the normal AFP was 5.2 ng ml$^{-1}$ (Maussier *et al.*, 1990). In cirrhotic patients with AFP values higher than 18.5 ng ml$^{-1}$ the likelihood of HCC being present is 95% (Maussier *et al.*, 1990). A cut-off value of 150 ng ml$^{-1}$ for diagnosis of HCC was suggested previously (Fujiyama *et al.*, 1992). The diagnostic level of AFP in China is 400 ng ml$^{-1}$ (Chen and Sung, 1977). As shown in this study, AFP levels less than 400 ng ml$^{-1}$ were found in 36.6% (37/101) of HCC patients at the time of tumour detection. It is obvious that AFP alone is not a reliable indicator for detection of HCC in patients with a low AFP level. Therefore, additional and more sensitive diagnostic tools must be sought. In this study, regardless of which cut-off value (120 ng ml$^{-1}$ or 400 ng ml$^{-1}$) was selected, AFP showed a good specificity (100%) and moderate sensitivity (67.3% or 63.3% respectively) and a high positive likelihood ratio (Table IV). Based on the selected optimal cut-off value by ROC curve analysis, 3% PEG-CICs showed a high specificity (100%) and moderate sensitivity (65.3%). However, determination of AFP and 3% PEG-CICs in parallel significantly increased the diagnostic accuracy (Table IV). Although each test may not have sufficient sensitivity, the simultaneous use of both tests may be highly discriminatory in the detection of HCC. However, the combined positive predictive value will not be different from parallel assays if each positive assay is deemed clinically positive (Table IV). Parallel detection of both markers increases the number of tests performed, and this must have cost implications. As the cost of determining 3% PEG-CICs is low, and the test is easy to perform, the assay for 3% PEG-CICs should be performed to improve the detection of HCC in AFP-negative cirrhotics.

Elevation of AFP may be seen in patients with 'active' liver disease (Chen and Sung, 1977; Sherlock and Dooley, 1993). CICs may also be increased in patients with chronic liver disease (Brown *et al.*, 1983, 1984; Coursaget *et al.*, 1986; Tsai *et al.*, 1990*a*,*b*, 1991, 1995*a*–*c*). Such elevations may have an important effect upon the specificity of the tests. In this study, none of the patients with cirrhosis alone had AFP or 3% PEG-CICs greater than the optimal cut-off points selected by ROC curve analysis.

The major aim of tumour marker estimation in HCC is as a means of early detection (surveillance), particularly in the cirrhotic population. The present analysis has looked at a population of patients with a histologically proven diagnosis of HCC. It may be assumed that many of these had advanced disease and thus a high proportion would have significantly elevated tumour marker levels. The high specificity and sensitivity attained might therefore be overestimating the value of these tests as a surveillance tool. This clearly requires further evaluation.

In conclusion, this study shows that addition of an assay for 3% PEG-CICs to AFP gives a significant improvement in detection of HCC in patients with cirrhosis. The optimal cut-off value for AFP in the diagnosis of cirrhotic HCC is 120 µg ml$^{-1}$. In addition, 3% PEG-CICs should be used as an adjunctive tool in the detection of HCC in cirrhotics negative for AFP.

### References

BROWN SE, STEWARD MW, VIOLA L, HOWARD CR AND MURRY-LYON IM. (1983). Chronic liver disease: the detection and characterization of circulating immune complexes. *Immunology*, **49**, 673–678.

BROWN SE, HOWARD CR, STEWARD MW, AJDUKIEWICZ AB AND WHITTLE HC. (1984). Hepatitis B surface antigen-containing immune complexes occur in seronegative hepatocellular carcinoma patients. *Clin. Exp. Immunol.*, **49**, 673–683.

CHUANG LY, HON WC, YANG ML, CHANG CC AND TSAI JF. (1994). Urinary epidermal growth factor receptor-binding growth factors in the tumors of the digestive tract. *Clin. Biochem.*, **27**, 485–489.

COURSAGET P, BARRES JL, TORTEY E, COTTY P, DIOP T, YVONNET B, SOW MT, MBOP S, DIOP B, BOCANNDE JE, POURCELOT L, DROP MI AND CHIRON JP. (1986). Persistence of circulating complexes between HBsAg and immunoglobulin M in sera of hepatitis B surface antigen positive patients suffering from liver cirrhosis or primary liver cancer. *Cancer Res.*, **46**, 1492–1494.

CHEN DS AND SUNG JL. (1977). Serum alpha-fetoprotein in hepatoceullar carcinoma. *Cancer*, **40**, 779–783.

FIELDS HA, MCCAUSTLAND KA, BRADLEY DW AND MAYNARD JE. (1982). Purification of acute phase anti-hepatitis A virus (HAV) IgM and development of an IgM solid-phase radioimmunoassay for the detection of HAV. *J. Immunol. Methods*, **51**, 149–157.

FUJIYAMA S, ISUNO K, YAMASAKI K, SATO T AND TAKETA K. (1992). Determination of optimum cutoff levels of plasma des-gamma-carboxy prothrombin and serum alpha-fetoprotein for the diagnosis of hepatocellular carcinoma using receiver operating characteristic curves. *Tumor Biol.*, **13**, 316–323.

FUST G, KAVAI M, SZEGEDI GY, MERETEY K, FALUS A, LENKEY A AND MISZ M. (1980). Evaluation of different methods for detecting circulating immune complexes. An interlaboratory study. *J. Immunol. Methods*, **38**, 281–289.

HOPF U, SCHEFER HF, HESS G AND MEYER ZUM BUSCHENFELDE KH. (1981). In vitro uptake of immune complexes by parenchymal and non-parenchymal liver cells in mice. *Gastroenterology*, **80**, 250–259.

JENG JE AND TSAI JF. (1991). Hepatitis C virus antibody in hepatocellular carcinoma in Taiwan. *J. Med. Virol.*, **34**, 74–77.

KASAHARA A, HAYASHI N, FUSAMOTO H, KAWADA Y, IMAI Y, YAMAMOTO H, HAYASHI E, OGIHARA T AND KAMADA T. (1993). Clinical evaluation of plasma des-gamma-carboxy prothrombin as a marker protein of hepatocellular carcinoma in patients with tumors of various sizes. *Dig. Dis. Sci.*, **38**, 2170–2176.

LEE HS, CHUNG YH AND KIM CY. (1991). Specificity of serum alpha-fetoprotein in HBsAg+ and HBsAg− patients in the diagnosis of hepatocellular carcinoma. *Hepatology*, **14**, 68–72.

McCARTHY D, GODDARD DH, PELL BK AND HOLBOROW EJ. (1981). Intrisically stable IgG aggregates. *J. Immunol. Methods*, **41**, 63–74.

MANAGER BJ, KRAPF FE, GRAMATZKI M, NUSSLEIN HG, BURMESTER GR, KRAULEDAT PB AND KALDEN JR. (1985). IgE containing immune complexes in Churg-Strauss vasculitis. *Scand. J. Immunol.*, **21**, 369–373.

MAUSSIER ML, VALENZA V, SCHINCO G AND GALLI G. (1990). AFP, CEA, CA19-9 and TPA in hepatocellular carcinoma. *Int. J. Biol. Markers*, **5**, 121–126.

NERI B. (1983). Circulating immune complexes as 'tumor marker' in hepatoma-bearing rats (Yoshida AH 130). *Immunol. Lett.*, **6**, 59–61.

SALINAS FA, WEE KH AND SILVER HKB (1983). Immune complexes and human neoplasia. *Biomedicine*, **37**, 211–218.

SHERLOCK S AND DOOLEY J. (1993). *Disease of the Liver and Biliary System*, pp. 503–531. Blackwell Scientific Publications: Oxford.

SIMONETTI RG, CAMMA C, FIORELLO F, POLITI F, D'AMICO G. AND PAGLIARO L. (1991). Hepatocellular carcinoma: a worldwide problem and the major risk factors. *Dig. Dis. Sci.*, **36**, 962–972.

SOX HC, BLATT MA, HIGGINS MC AND MARTON K. (1989). *Medical Decision Making*, pp. 67–146, Butterworth: London.

SWETS JA. (1988). Measuring the accuracy of diagnostic systems. *Science*, **240**, 1285–1293.

TABOR E. (1991). Circulating immune complexes in hepatocellular carcinoma. *Cancer Invest.*, **9**, 241–242.

THOMAS HC, MACSWEEN RN AND WHITE RG. (1973). The role of the liver in controlling the immunogenicity of commensal bacterial bacteria in the gut. *Lancet*, **1**, 1288–1291.

TSAI JF, TSAI JH AND CHANG WY. (1990*a*). Relationship of serum α-fetoprotein to circulating immune complexes and complements in patients with hepatitis B surface antigen-positive hepatocellular carcinoma. *Gastroenterol. Jpn*, **25**, 388–393.

TSAI JF, MARGOLIS HS, FIELDS HA, NAINAN OV, CHANG WY, TSAI JH. (1990*b*). Immunoglobulin and hepatitis B surface antigen-specific circulating immune complexes in chronic hepatitis with hepatitis delta virus infection. *J. Med. Virol.*, **30**, 25–26.

TSAI JF, TSAI JH, CHANG WY, TON TC. (1991). Elevation of circulating immune complexes and its relationship to α-fetoprotein levels in patients with hepatitis B surface antigen-positive hepatocellular carcinoma. *Cancer Invest.*, **9**, 137–143.

TSAI JF, CHANG WY, JENG JE, WANG LY, HSIEH MY, CHEN SC, CHUANG WL AND LIN ZY. (1993). Hepatitis C virus infection as a risk factor for non-alcoholic liver cirrhosis in Taiwan. *J. Med. Virol.*, **41**, 296–300.

TSAI JF, CHANG WY, JENG JE, HO MS, LIN ZY AND TSAI JH. (1994*a*). Hepatitis B and C virus infection as risk factors for liver cirrhosis and cirrhotic hepatocellular carcinoma: a case–control study. *Liver*, **14**, 98–102.

TSAI JF, CHANG WY, JENG JE, HO MS, LIN ZY AND TSAI JH. (1994*b*). Frequency of raised alpha-fetoprotein level among Chinese patients with hepatocellular carcinoma related to hepatitis B and C. *Br. J. Cancer*, **69**, 1157–1159.

TSAI JF, JENG JE, HO MS, CHANG WY, LIN ZY AND TSAI JH. (1994*c*). Hepatitis B and C virus infection as risk factors for hepatocellular carcinoma in Chinese: a case–control study. *Inter. J. Cancer*, **56**, 619–621.

TSAI JF, JENG JE, CHANG WY, LIN ZY AND TSAI JH. (1994*d*). Hepatitis C virus infection among patients with chronic liver disease in an area hyperendemic for hepatitis B. *Scand. J. Gastroenterol.*, **29**, 550–552.

TSAI JF, CHANG WY, JENG JE, HO MS, LIN ZY AND TSAI JH. (1994*e*). Effects of hepatitis C and B viruses infection on the development of hepatocellular carcinoma. *J. Med. Virol.*, **44**, 92–95.

TSAI JF, MARGOLIS HS, JENG JE, HO MS, KO YC, CHANG WY, LIN ZY AND TSAI JH. (1994*f*). Association between hepatitis B and C virus infection and Chinese hepatocellular carcinoma: a case–control study. In *Viral Hepatitis and Liver Disease*, Nishioka K, Suzuki H, Mishiro S and Oda T (eds) pp. 697–700. Springer: Tokyo.

TSAI JF, JENG JE, CHANG WY, HO MS, LIN ZY AND TSAI JH. (1995*a*). Circulating immune complexes in chronic hepatitis C. *J. Med. Virol.*, **46**, 12–17.

TSAI JF, JENG JE, CHANG WY, HO MS, LIN ZY AND TSAI JH. (1995*b*). Increased IgM-containing circulating immune complexes in patients co-infected with hepatitis C and hepatitis B. *Medicine*, **74**, 136–143.

TSAI JF, MARGOLIS HS, JENG JE, HO MS, CHANG WY, LIN ZY AND TSAI JH. (1995*c*). Circulating immune complexes in chronic hepatitis related to hepatitis C and B viruses infection. *Clin. Immunol. Immunopathol*, **75**, 39–44.

ZUBLER RH, PERRIN LH, CREIGHTON WD AND LAMBERT PH. (1977). Use of polyethylene glycol to concentrate immune complexes from serum or plasma samples. *Ann. Rheum. Dis.*, **36** (Suppl.), 23–26.